# An Insight into Biomolecules for the Treatment of Skin Infectious Diseases

**DOI:** 10.3390/pharmaceutics13071012

**Published:** 2021-07-02

**Authors:** Helena P. Felgueiras

**Affiliations:** Centre for Textile Science and Technology (2C2T), University of Minho, Campus de Azurém, 4800-058 Guimarães, Portugal; helena.felgueiras@2c2t.uminho.pt; Tel.: +351-253-510-289

**Keywords:** pathogenic microorganisms, disruption of skin functions, biomolecule-based treatments, mechanisms of action, prevalent skin infectious diseases

## Abstract

In assigning priorities, skin infectious diseases are frequently classified as minor when compared to infectious diseases of high mortality rates, such as tuberculosis or HIV. However, skin infections are amongst the most common and prevalent diseases worldwide. Elderly individuals present an increased susceptibility to skin infections, which may develop atypical signs and symptoms or even complicate pre-existing chronic disorders. When the skin fails to correct or inhibit the action of certain pathogenic microorganisms, biomolecules endowed with antimicrobial features are frequently administered topically or systemically to assist or treat such conditions. (1) Antibiotics, (2) antimicrobial peptides, or (3) natural extracts display important features that can actively inhibit the propagation of these pathogens and prevent the evolution of infectious diseases. This review highlights the properties and mechanisms of action of these biomolecules, emphasizing their effects on the most prevalent and difficult to treat skin infections caused by pathogenic bacteria, fungi, and viruses. The versatility of biomolecules’ actions, their symbiotic effects with skin cells and other inherent antimicrobial components, and their target-directed signatures are also explored here.

## 1. Introduction

Infectious diseases are emerging or re-emerging almost every year. They are a result of the presence and growth of pathogens, such as viruses, bacteria, parasites, and/or fungi, in the host organism, which can propagate between people. The type and extent of damage inflicted by these microbial agents, which is intimately related to the production and liberation of toxins that affect the regular tasks of each organ/system, could lead to the appearance of specific clinical symptoms [1,2].

Since the beginning of the 20th century, infectious diseases have been classified as the primary cause of death worldwide. Indeed, emerging infectious diseases are rising [2,3]. This trend is expected to continue, as newly evolved strains of pathogens emerge (multi-resistant) and enter the human population without a proper fighting response being available (e.g., severe acute respiratory syndrome (SARS) coronavirus). The increase in the global population and its aging, the climate changes, and the facility of travel between countries have all favored the emergence, evolution, and spread of these new pathogens [4]. Projection patterns of the evolution of this crisis have highlighted bacteria and viruses as the most prevalent and concerning group of pathogens: the first due to their increasing resistance to antibiotics and the second due to their high capacity to adapt to new hosts.

Skin infections are amongst the most common infectious diseases, ranked as the fourth leading cause of human illnesses [5]. Both bacteria and viruses are responsible for many serious, difficult to treat skin conditions. Fungi are also highly prevalent in skin diseases. Skin infections present considerable threats to a person’s health, psychological wellbeing, capacity to operate, and social involvement. In 2013, the socioeconomic burden associated with skin diseases was estimated at USD 75 billion and USD 11 billion for direct (healthcare expenses) and indirect (professional constraints) costs, respectively, in the USA [6]. As the population ages, the amount of people affected by skin conditions rises proportionally. Tears in the skin integrity, which become more frequent with age as the tissue loses its inherent elasticity and flexibility, are frequently inoculated by pathogens that invade the dermis, causing skin infections. In many situations, the skin microbiota and the antimicrobial factors produced by the various cell layers forming this tissue are capable of fighting infections and preventing serious illnesses from evolving [7]. However, as the pathogens evolve and mutate and the population ages, our inherent immune defenses become compromised, being unable to effectively respond in a timely manner to their action. Antimicrobial agents based on biological cues are, in many instances, essential to overcome the shortcomings of our immune system and assist in the treatment of infectious diseases [8]. Indeed, there are many antimicrobial biomolecules currently available to fight pathogenic microorganisms that affect the skin. Antibiotics, due to their effectiveness and accessibility, are among the most prevalent biomolecules to be used in treatments of infectious diseases. They target the physiology or the biochemistry of the microorganism, inhibiting their replication or inactivating specific functions, ultimately leading to their death. Yet, due to the appearance and propagation of resistant microbial cells, therapies for skin infections have become more challenging in the last twenty years [8,9]. New biomolecules that impart specific cell functions or leave a smaller environmental footprint are now raising more attention [10]. In the present work, an overview of the skin inherent mechanisms of action against microbial cells and the alternative biomolecules that may be used to assist in this action or surpass its limitations is presented. A complete description of the most prevalent skin infectious disease traits and the possible biomolecules that can be or are already being applied to treat such conditions is also provided, where special emphasis is placed on natural-origin biomolecules. Generally, this publication reviews the most recent research that explores the use of conventional or recently emerging biomolecules to treat skin infections.

## 2. Skin: The First Line of Defense

**Figure 1:**
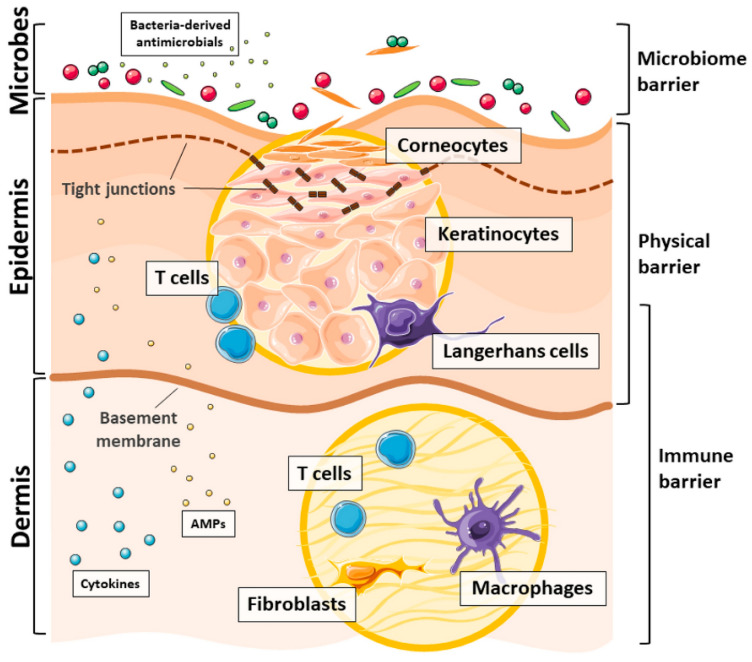
Division of the skin and its mechanisms of protection against microbial penetration [13]. (CC BY 4.0 license).

The skin is humans’ largest and most exposed organ, offering protection against the loss of endogenous compounds and external stimuli, including microbial invasion (Figure 1). It forms 8% of the body weight and is subdivided into three major layers, the epidermis which is the most superficial layer, the dermis located beneath the epidermis and made mostly of connective tissues, and the hypodermis, which is a deeper, subcutaneous tissue made from connective and adipose tissues [7,11,12].

The protective features of the skin vary across its regions and the age of the person, often generating conditions conducive for microbial growth in localized microenvironments or exhibiting properties that impart a direct antimicrobial protection. Microorganisms present along the skin are one of the largest communities of microbes in humans. Indeed, the cutaneous microbiota contains bacteria, fungi, and viral communities. These resident microbial species defend the host from pathogenic organisms by producing antimicrobial factors or stimulating cells to secret antimicrobial cues that prevent the growth and proliferation of pathogens [14,15]. For instance, the skin-resident coagulase-negative *Staphylococci* bacteria can produce toxins such as phenol-soluble modulins that attack more harmful strains of bacteria, such as *Staphylococcus aureus* or *Streptococcus pyogenes*. Further, they can work in symbiosis with the skin antimicrobial peptides (AMPs) to inhibit pathogen colonization [7].

The epidermis operates as the first line of protection against microorganisms. It is formed mostly of keratinocytes, whose phenotype and functions differ along the stratified epidermis, the stratum corneum, the stratum granulosum, the stratum spinosum, and the stratum basale. These keratinocytes express Toll-like receptors (TLRs) that trigger pathways that maintain the host–microbial homeostasis [16]. These stratums offer resistance against the penetration of most microbes by engaging with AMPs to fortify the integrity of the skin barrier. Indeed, the epidermis is formed mostly of AMPs and lipids, both exhibiting antimicrobial functions. AMPs are small peptide molecules that can both kill or inhibit microbial responses, limiting their growth. Their secretion is dependent on the conditions of the skin (healthy or infected) [17]. Antimicrobial lipids, in their turn, are synthesized and transferred to the surface of the skin during cell differentiation, being capable of fighting Gram-positive and Gram-negative bacteria and increasing the expression of pro-inflammatory cytokines to signal infections [18].

At the dermis, fibroblasts produce collagen, elastin, and other extracellular species while still increasing the skin antimicrobial signals and, in this way, working as immunomodulatory cells [19]. The dermis fibroblasts produce cytokines (interleukin (IL)-6, IL-8, tumor necrosis factor alpha (TNFα)), growth factors (vascular endothelial growth factor (VEGF), transforming growth factor beta 1 (TGFβ1)), and metalloproteinases (MMP-1) that stimulate the adhesion and migration of immune cells, such as macrophages, mast cells, leukocytes, and dermal dendritic cells [7]. Even though the antimicrobial action of the dermis is not as important as that of the epidermis, the infiltration and recruitment of immune cells to fight microbial pathogens that occur in this layer remain vital. These cells provide additional antimicrobial defense by promoting the liberation of large amounts of cathelicidins, such as the AMP LL37 [20], that not only fight microbial colonization but also stimulate keratinocyte proliferation and induction of angiogenesis. Indeed, in injured, infected portions of the skin, the dermal fibroblasts and the epidermal keratinocytes work in synergy to restore the skin barrier [19].

The hypodermis, also known as the subcutaneous layer, has a small but still very important part to play in protecting the skin against microbial infection. The adipose tissue of the subcutaneous layer can be subdivided into brown adipose tissue (BAT), white adipose tissue (WAT), and “beige” adipose tissue [21]. The WAT, which can be found close to hair follicles, altering in size as the hair grows, or underneath the dermal layer, is also capable of producing cathelicidin AMPs and expands in size for a quick proliferation of adipocyte precursors. A recent study reported a causal relationship between the expansion of adipose tissues and the hypodermis’s ability to protect against pathogenic cells [7,22]. This protective behavior inherent to all layers composing the skin relies on their cell components or immune cells to which they impart a specific function, by meticulously triggered pathways. Still, many pathogenic microbes can disrupt this specialized line of defense, acting quickly against the human body and causing serious skin infections. To overcome these shortcomings, antimicrobial biomolecules are frequently employed.

## 3. Biomolecules: Types, Properties, and Mechanisms of Action against Pathogens

Biomolecule is a loosely applied term to define biological materials derived from living organisms (animals, plants, etc.) that exert important tasks in specific biological phenomena. In infectious diseases, these biological cues are usually administered orally or immobilized/incorporated within delivery systems for a local or systemic distribution [23]. In skin infections, three major classes of biomolecules with antimicrobial potential have been identified: antibiotics, AMPs, and natural extracts.

### 3.1. Antibiotics

Antibiotics are considered the greatest clinical advance of the 20th century. They are defined as chemical substances of microbial or synthetic origin endowed with antimicrobial features that can treat infectious diseases. In order to be categorized as effective, antibiotics should target intact elements of the bacterial cell wall, should reach their target in sufficient amounts to elicit the desired effect, and should not undergo chemical alterations that may hinder their effectiveness [24]. Antibiotic action can be classified as bactericidal, killing bacteria, and/or bacteriostatic, preventing bacterial growth. Bactericidal antibiotics, such as glycopeptides, beta-lactams, or fluoroquinolones, are often preferred to bacteriostatic antibiotics in the treatment of skin disorders. These target the cell membrane of bacteria, the cell wall biosynthesis of enzymes and other substrates, protein synthesis, or bacterial nucleic acid replication, compromising the cell integrity and eventually leading to its death [25]. Their specific structure and metabolism enable them to act against pathogenic microbials with minimal side effects to mammalian cells [8].

Alterations in their performance may lead to bacterial resistance. This may occur in response to: target modifications through mutation, decreased bacteria cell permeability, reduced biomolecule influx or increased efflux, and degradation/deactivation of the antibiotic molecules by means of enzymatic hydrolysis or chemical action [26]. The overuse or misuse of these biomolecules has increased bacteria tolerance and the occurrence of antibiotic-resistant strains. Indeed, the spread of resistant bacteria has become a growing public health concern, which renders lifesaving drugs less effective. For that reason, alternative therapies based on AMPs or natural extracts are now gaining more impact in patient care. Still, many efforts continue to be devoted to the development of new and more effective drugs. Despite these drawbacks, antibiotics remain, to this day, the main source of antimicrobial agents to fight bacteria-derived skin infections. Newer, directed-spectrum antibiotics are, in fact, being design to circumvent these multidrug resistance mechanisms [8,10].

### 3.2. Antimicrobial Peptides

AMPs are progressively gaining more focus as new therapies to treat skin infections. Indeed, one of their pivotal roles in the skin is to guarantee its innate immunity. Their significance in skin treatments became particularly noticeable when the release of cathelicidins during wound healing was uncovered and when the presence of β-defensins in psoriatic scales was determined ample. AMPs not only impart antimicrobial effects but are also involved in triggering immunological responses against microbial pathogens [27].

**Figure 2:**
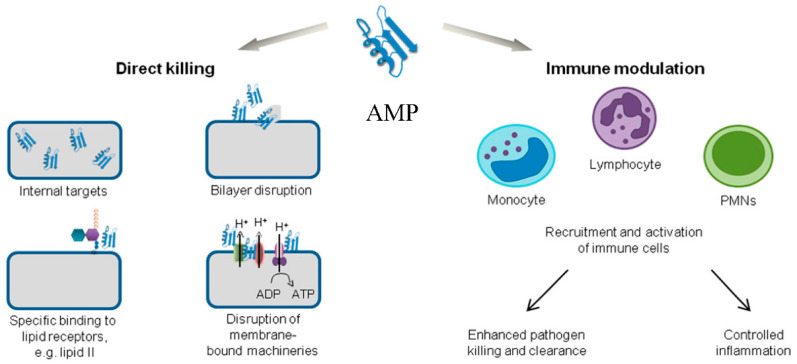
Representation of the main mechanisms of action of antimicrobial peptides [30]. (CC BY 4.0 license).

AMPs are oligopeptides formed of short sequences of amino acids. AMPs are low-molecular weight biomolecules, cationic, and amphiphilic, which can be chemically synthesized or secreted by procaryotic and eukaryotic cells (e.g., human skin). To date, all identifiable AMPs are categorized in one of four categories, according to their secondary structure: β-sheet, α-helix, extended, and loop, with the first two configurations being the most common. They target the lipopolysaccharide layer of microorganisms, which is unique to them, hence expressing little effects against the low-anionic and cholesterol-rich human eukaryotic cells. Moreover, due to their quick killing effect, resistance by microorganisms is more difficult to cultivate. Depending on their target microbial agent, they can be classified as antibacterial, antifungal, antiviral, or antiparasitic. In all cases, AMPs act against the microbial cells by disrupting the membrane/wall/envelope integrity, inhibiting proteins’, nucleic acids’, or other important compounds’ synthesis, or by permeabilizing the microorganism and interacting with certain intracellular targets (Figure 2). One of the most important features of AMPs is their ability to act against microbial cells using a multi-hit strategy based on non-specific mechanisms of action, which tends to broaden their spectrum of antimicrobial action even against multi-resistant pathogens [8,17,28,29].

Recent studies have reported evidence of resistance mechanisms by pathogens against naturally occurring skin AMPs. To overcome this obstacle, synthetic peptides are being engineered. Research has shown that even very structurally similar peptides, synthesized based on known natural structures, can impart completely different mechanisms of action and even increase their range of targeted microbials. Another option has been to combine different origin biomolecules, such as antibiotics with AMPs or AMPs with herbal derivatives, to fight the enhanced microbial action of these resistant pathogens [31].

### 3.3. Natural Extracts

**Figure 3:**
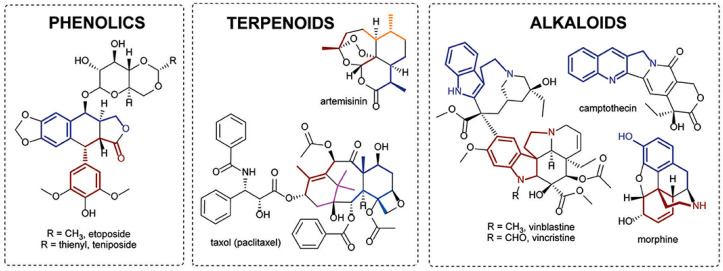
Major classes of natural extracts, phenolics, terpenes or terpenoids, and alkaloids (adapted with permission from [34]). (CC BY 4.0 license).

A substantial percentage of extracts collected from plants are claimed to display beneficial results in infection control. To date, more than 12,000 secondary plant metabolites have been isolated and identified, a number that represents less than 10% of the total possibilities still available and to be discovered. These secondary metabolites act as antioxidants, free radical-scavenging actuators, UV light absorbents, or protectors against potential environment-derived attackers, namely, microorganisms. The palette of secondary metabolites can be subdivided according to the chemical structure and synthetic pathways of their active compounds. The major classes encompass (Figure 3): (1) phenolics, whose structural motif is the phenol molecule, and which are produced by plants in response to bacterial and fungal attacks against which they can generate non-specific interactions with proteins or inhibit enzyme actions; (2) terpenes, whose structure includes one or more five-carbon isoprene units, and which display toxicity that varies from lethal to completely comestible depending on their ecological roles and may act against microbial pathogens by disrupting their membrane through lipophilic substances; and (3) alkaloids, which are heterocyclic nitrogen complexes able to intercalate with microbial pathogens’ DNA, compromising their normal functions [8,32]. Within the terpenes class, it is possible to find a very important category of plant extracts that has been gaining considerable attention, namely, essential oils (EOs). These are aromatic, volatile substances that can impart inhibitory or biocidal effects against the replication of many microbial cells. EOs can permeate the cell membrane, reducing the presence of important intracellular components by raising the local acidity, which blocks the production of ATP and destroys genetic material [33]. Even though their toxicity to human cells has been a major limitation to their generalized application in medicine, with allergic reactions and skin irritation being the most recurrent side effects, recent studies in which nano- and micro-delivery vehicles have been employed or the EOs proportion has been reduced in combination with other antimicrobial substances have been very promising, suggesting an optimistic future for EOs-containing strategies to treat skin problems [10].

## 4. Prevalent Skin Infections and the Biomolecules Employed in Their Treatment

Skin infections can affect the skin layers and the inherent connective tissues. From the neonate to the elderly, all of the human population can be affected by a wide spectrum of skin diseases. In fact, when reaching 70 years old, nearly 70% of people will have had a minimum of one skin condition in their life [35]. Bacteria, viruses, and fungi are amongst the microbial pathogens responsible for the most frequent skin infections. In this section, special attention will be placed on those dermatological illnesses that may benefit from the action of conventional and/or new biomolecules in their treatments, namely, antibiotics, AMPs, and natural extracts.

### 4.1. Atopic Dermatitis

Atopic dermatitis (AD) is a very frequent inflammatory disorder, highly pruritic, and related to cutaneous hyper-reactivity to triggers present in the environment. It is identifiable by its poorly delineated erythema with edema, presence of vesicles, and, in acute states, chronic weeping and skin thickening. There has been much debate as to whether AD is an allergen-induced disease, or an inflammatory skin disorder found in combination with respiratory conditions. Regardless, investigations have demonstrated that AD is characterized by an intricate etiology, with the stimulation of various immunologic and inflammatory pathways. The prevalence of this condition is superior in children, with 10–20% of children being affected compared to only 1–3% of adults [36]. Due to the industrial revolution of the last three decades and the increased worldwide levels of pollution, these rates have been continuously increasing, with effective treatments becoming highly demanded [37].

AD is typified by dry skin and enhanced transepidermal loss of water. In AD, ceramides, which are lipids present in the skin layers responsible for conserving the molecules of water, are in deficit. Thus, the skin becomes more susceptible to irritants, conditioning epidermal differentiation and prompting inflammatory responses. Allergens may also trigger scratching of the skin which initiates the inflammatory cascade by releasing pro-inflammatory cytokines from atopic keratinocytes [38]. In most patients with AD, as the skin barrier becomes compromised, infections occur. *S. aureus* is the most prevalent microorganism. It tends to aggravate AD conditions by producing toxins known as superantigens that activate T cells and macrophages as the patients try to secret immunoglobulin E (IgE) antibodies, specific against these molecules. They also induce corticosteroid resistance, raising the severity of the patient’s condition. As *S. aureus* binds to the inflamed skin, its innate response becomes compromised as this bacterium prevents the skin components from producing AMPs necessary to protect the host from bacteria, fungi, and virus colonization. For that reason, people suffering from AD have an enhanced tendency to develop infections spread by herpes simplex or vaccinia viruses, for instance [36,39,40].

The conventional treatments for AD-related infections rely on the topical administration of antibiotics. Fusidic acid and mupirocin are among the most common. However, recent reports have suggested that bacteria are starting to develop resistance. Alternatively, formulations that combine gentamycin with corticosteroids have become more popular in AD treatments. Another option is to employ antiseptics, such as triclosan or chlorhexidine, in the form of emollients, which can significantly reduce colonization by *S. aureus* without significant risks of skin sensitization. In cases where infection is caused not only by *S. aureus* but also by *S. pyogenes*, systemic antimicrobials are preferable. Here, amoxicillin/clavulanate or clindamycin antibiotics may be administered for a period of 3 to 7 days, even though gastrointestinal discomfort may occur. For longer periods, courses of sulfamethoxazole/trimethroprin are recommended to prevent the development of microbial resistance [41]. In recent years, antibody- or cytokine-directed therapies have been uncovered with successful outcomes in preventing T cell activation or the over-secretion of cytokines and other mediators involved in AD. For instance, omalizumab, a humanized monoclonal antibody selectively directed against circulating IgE, may block circulating IgE’s interaction with the receptors of the basophils and the mast cells’ membrane, inhibiting their activity, degranulation, and the liberation of different mediators responsible for AD [42]. Even though these inhibitor-based therapies are very sought after, their intervention against infection agents is considered minimal. Indeed, in many cases, additional or secondary therapies are recommended. Herbal preparations are often used. Aloe vera, for instance, has shown significant effects on AD skin lesions by reducing IL-5 and IL-10 levels while fighting the presence of bacteria and fungi [43]. *Cannabis sativa* is also employed to treat itchiness and relieve pain symptoms in AD. Using the oil from the seeds of this plant, it is possible to strengthen the skin, increasing its resistance to bacterial, viral, and fungal infections [44]. German chamomile (*Matricaria recutita*) has been used for centuries to treat skin inflammations, including dermatitis. Topical chamomile ointments have even been compared to hydrocortisone-containing formulations for their ability to reduce the affected surface area or by improving sodium lauryl sulfate-induced contact dermatitis. Their antimicrobial and anti-inflammatory performance has been attributed to the presence of an essential blue oil in their composition, which is formed of sesquiterpene alcohol, α-bisabolol, chamazulene, and flavonoids [45]. Tannin agents derived from black tea or oak bark have also been suggested as effective in relieving acute weeping, or in soothing inflammation and skin pruritus. They act by coagulating the protein regions in the cell surface and the exudates, thus reducing absorbency and secretion, and by acting against pathogenic microbials, reducing infection [46].

Flaws in the AMPs present in the skin barrier may contribute to the AD pathogenesis. Indeed, LL37, hBD-2, and hBD-3 AMPs are frequently downregulated in lesions caused by AD. In many instances, this reduced AMP expression is even triggered by the use of corticosteroids and calcineurin inhibitors applied topically [47]. To overcome such problems, many therapeutics targeting the skin microbiome and the inherent AMP expression have been formulated with the goal of restoring skin homeostasis and, thus, overturning AD symptoms. Smits et al. suggested the use of coal tar to activate the receptor aryl hydrocarbon and, in this way, induce the secretion of AMPs derived from keratinocytes. Here, as coal tar reduced the abundance of *S. aureus* and restored the microbiota composition to healthier rates, the AMP levels in AD skin were also elevated, rendering the affected area less prone to infection and inflammation [48].

### 4.2. Psoriasis

Psoriasis is an autoimmune chronic inflammatory skin condition in which localized lesions, demarcated erythematous, and silver scaly plaques are often observed on the knees, elbows, trunk, and scalp. It is characterized by an abnormal keratinocyte proliferation and differentiation, increased dermal blood vessel formation with enhanced permeability of wide-caliber vessels, and dermis invasion by several immune cells which increase the inflammation mediated by cytokines, including tumor TNFα, interferon-γ (IFN-γ), and various ILs [49]. The prevalence of this skin infectious disease has been rising over time, now affecting 2–3% of the global population, with 0.5–11.4% being adults and 0–1.4% being children [50]. In patients with psoriasis, the life cycle of the skin reduces from 30 to 1.5–3.0 days, which facilitates the appearance of localized lesions [51]. Dry, cracked skin and irritation are also very common. Psoriatic areas may vary in size from a few spots of dandruff-like scaling to large outbreaks that spread significantly across the body. Various forms of psoriasis have been identified: (1) plaque, the most frequent, characterized by red skin patches and silvery scales that predominate on the elbows, the lower back, the knees, or the scalp; (2) nail, which affects fingernails and toenails, resulting in pitting, atypical nail development, and discoloration; (3) guttate, which is primarily detected in children and young adults and is triggered by bacterial infections; (4) inverse, which causes the appearance of smooth red skin patches on intimate regions (e.g., groin, breasts, or buttocks) and may be aggravated with the friction and sweat sensed on these areas; (5) pustular, which is more rare and is characterized by the appearance of pus-filled lesions; (6) erythrodermic, the least common, which affects the entire body of the patient, covering it with a red, peeling rash; and (7) arthritis, which affects the patient’s joints and may cause stiffness or progressive joint damage [52].

Many microorganisms have been associated with the initiation and/or worsening of psoriasis, regardless of the type, including bacteria (*S. pyogenes*, *S. aureus*), fungi (*Malassezia*, *Candida albicans*), and viruses (papillomaviruses, retroviruses, endogenous retroviruses) [53]. The most significant effect has been that of the bacterium *S. pyogenes*, evidenced in both acute and chronic psoriatic patients. This pathogen is mostly associated with the guttate form of psoriasis. Here, superantigens or T cell-activating toxins generated by this bacterium can bind to the T cell receptors, inducing the manifestation of a skin homing receptor, the cutaneous lymphocyte antigen, which may trigger an autoimmune reaction on the skin [54]. Near 60% of psoriatic patients present lesions colonized by *S. aureus*. The presence of toxin-positive *S. aureus* on patients’ skin was found to have a significant impact on the dimensions of the affected area and the severity of the disorder [55]. In the case of fungi, *Malassezia* tends to occupy the outermost regions of the stratum corneum rich in sebaceous glands, frequently being found associated with lesions present on the chest, back, and scalp. They act by upregulating keratinocyte expression, which then results in a hyperproliferation and cell migration of the epidermis [52,56]. The mechanism by which *C. albicans* exacerbates psoriasis is not yet known. However, a correlation with an increased number of this pathogenic yeast’s presence in feces of psoriasis patients has been established. It is suspected that *C. albicans* may produce superantigens just like *S. pyogenes* or *S. aureus*, stimulating T cell activation and, thus, leading to an increased severity of the psoriatic lesions [52].

Currently, psoriasis does not have a cure. Yet, many strategies have been designed to try and relieve the patient symptoms and prevent the evolution of psoriasis-related signs. Conventional topical medications incorporate corticosteroids, vitamin D3, retinoids, calcineurin inhibitors, coal tar, dithranol, and emollients, while systemic therapies frequently resort to immunomodulators (cyclosporine) and retinoids (acitretin). Corticosteroids are considered the gold standard for psoriasis treatment. Once applied, corticosteroids can bind to the glucocorticoid receptors in the cells’ cytoplasm, increasing the formation of lipocortin, which is responsible for inhibiting the phospholipase A2 activity and the formation of IL-1. These actions wield anti-inflammatory, immunosuppressant, anti-proliferative, and anti-mitogenic impacts. However, they may also affect the epidermis, rendering the skin photosensitive, delaying wound healing, and causing ulceration or even atrophy of the affected areas [57]. To prevent such events, emollients and moisturizers, which exhibit anti-inflammatory effects, prevent microbial invasion, and reduce transepidermal water loss, may be used as adjuvant actuators. The use of these antibiotics in psoriasis has been prompted mostly by the presence of streptococci bacteria in different forms of the disease. They act against the bacteria by preventing the colonization of pathogens along the lesions or by targeting specific factors associated with their infectious potential. Even though they remain a highly effective choice, they may also hinder skin healing or even exacerbate the condition (Table 1) [58]. In recent years, biologics, which are biomolecules that target the immune or genetic mediators in a pathophysiologic event, have been used to target T cells or block the action of TNFα and IL actuators and, in this way, reduce the inflammatory response in psoriasis. The USA Food and Drug Administration (FDA) endorsed the first biologics in 2003, namely, alefacept and efalizumab, but since then, many more have progressed successfully through clinical trials and have reached the market. These biomolecules have been considered more effective than corticosteroids as they suppress the activated local immune response. However, their long-term use may increase the risk of uncontrolled infections [51,59]. Recent research has demonstrated that, by combining these biologics with metabolites present in vitamins, it is, indeed, possible to overcome such problems. For instance, the use of IL-17 inhibitors, which modify the cytokine pathways, may also increase the risk of fungal infections caused by *Candida* spp. Vitamin A is a fat-soluble vitamin involved in a variety of metabolic and physiologic activities but also works as a host-protective effector against bacterial, viral, and yeast infections. Since IL-17 is engaged in the inflammatory reaction responsible for triggering and sustaining psoriatic disease, and knowing that TH17 cells specific to *C. albicans* produce IL-17, combinations of inhibitors of this cytokine with antimicrobial molecules are seen as a promising solution [60].

For many years, natural extracts have been used to treat infected skin lesions in psoriasis, mostly as a complementary therapy. Capsaicin (*Capsicum frutescens*) was determined to hamper phorbol ester-stimulated initiation of transcription factors NF-κB and AP-1, which then resulted in a significant decrease in scaling and erythema in moderate to severe psoriasis patients [61]. Curcumin behaves similarly, being capable of suppressing the phosphorylase kinase activity and even imparting antimicrobial and antioxidant effects onto the affected areas. In the form of nano-emulsions or while microencapsulated, its action is even more effective, reducing the yellow staining of the skin derived from its topical administration [62]. EOs can provide soothing effects to the affected areas of the skin and even effectively irradiate pathogens. Tea tree oil (TTO), rose geranium oil, and lavender oil are all endowed with anti-inflammatory features as well as antibacterial and antifungal properties. Marine sponges, such as *Dysidea avara*, are also known to effectively inhibit inflammatory biomarkers, such as TNFα, and to suppress the action of keratinocytes. Most importantly, they are effective antiviral biomolecules and may display moderate antibacterial activities [63].

### 4.3. Herpes Simplex and Zoster

Herpes simplex viruses type 1 and type 2 (HSV1 and HSV2) and varicella-zoster virus (VZV) are neurotropic pathogenic viruses found in humans. Both HSV and VZV are affiliated to the herpesviridae DNA viruses. The first is frequently detected in the labial skin of the mouth (HSV1) or genitalia (HSV2), and the second is characterized by cutaneous eruptions along the skin filled with pus that tend to rupture and scab before healing. HSV1 is mostly transmitted by oral–oral contact but may also be transmitted by oral–genital contact. Oral herpes infection is mostly asymptomatic. However, those that exhibit symptoms tend to experience painful blisters or open sores in or around the mouth. HSV2 is mostly transmitted during sex, through genital contact. As with oral herpes, people affected by HSV2 are often asymptomatic, and those exhibiting symptoms reveal the presence of blisters or open sores around the genitals or anal region. Both HSV and VZV share specific identifiable traits that influence their infective sequence in the body: they start by acting on the mucocutaneous regions, which work as an entryway portal of infection, and then may be transported towards the dorsal root ganglia, where they are kept in a latent form until the virus is activated. Even though primary infection occurs in the first twenty or thirty years of a person life’s, revival of the virus may still take place in advanced ages [64,65].

Antivirals such as acyclovir, famciclovir, and valacyclovir are very effective in treating HSV. They act by preventing viral replication by means of inhibiting DNA polymerase action in infected cells. The application of corticosteroids as secondary or additional therapies remains contentious; however, when HSV is associated with encephalitis conditions, a high dose of steroids may be recommended [64]. *Melissa officinalis* oils presented to HSV1 lesioned areas in the form of balms have been shown to significantly act on patients by reducing the size of the ulcerations and the healing time. Here, tannin and polyphenols have been indicated as responsible for the antiviral effects of these balms [66]. These same antivirals may also be used to treat or prevent HSV2. However, a prophylactic and corrective anti-HSV2 vaccine is usually recommended.

VZV is mostly a disorder of older people, and its appearance may be triggered by disorders such as diabetes mellitus, spinal anesthesia, malignancies, and conditions associated with immune suppression (e.g., AIDS) [64]. The therapeutic approach is similar to HSV, resorting mainly to the antivirals acyclovir, famciclovir, and valacyclovir, the last two being preferrable due to their superior pharmacokinetic reports and easier dosing routines. Alternatively, the antiviral drugs brivudine and foscarnet may also be employed [67]. The natural extracts *Capiscum frutescens* and hibiscus have been reported to reduce localized pain generated by ulcers, while glycyrrhizin-derived products were reported to hinder the in vitro propagation of varicella zoster [68,69]. At the moment, no in vivo studies have been conducted using these natural components.

### 4.4. Acne

Acne vulgaris is a very frequent skin condition. It often appears in oily regions of the body, namely, the face, back, and trunk. Acne vulgaris is considered an inflammatory disease of pilosebaceous follicles, affecting over 85% of the youngster population. It is caused by a heightened sebum formation, hypercornification of the pilosebaceous duct, abnormal behavior of the microbial skin flora with colonization of the duct with *Cutibacterium acnes,* and an irregular inflammatory response. Even though acne is not considered an infectious disease per say, four key microorganisms can be isolated from the skin of patients suffering from this disorder, namely, C. acne, *Cutibacterium granulosum*, *Staphylococcus epidermidis*, and *Malasezia furfur.* These are normal skin commensals that tend to proliferate very quickly during puberty, very often being responsible for the development of acne. Indeed, the hypercolonization of the sebaceous follicle by C. acnes has been known to increase acne-related inflammation by inducing immunological reactions, including the upregulation of cytokine expression by its extracellular biproducts, the sebum free fatty acids metabolized by the triglycerides. Contrary to the other microbials, S. epidermidis is regularly engaged in superficial infections within the sebaceous unit [70,71,72].

For many years, topical retinoids, benzoyl peroxide, and topical/systemic antibiotics have been used to treat this condition. Lymecycline, erythromycin, and oxytetracycline are among the most common antibiotic strategies [73]. However, *C. acnes* has been revealing increased resistance to these standard therapies. It is not yet clear if the resistance to the action of the antibiotics is indeed related to the presence of this microbial cell or the combination of various pathogens with endogenous unregulated factors. There have been reports showing variations in response to antibiotics from *C. acnes* colonies within the same culture. Differences between *C. acnes* strains with the ability to form biofilms resistant to antimicrobials have also been described. For instance, beta-lactam antibiotics are ineffective against acne, but quite effective against in vitro cultures of *C. acnes* [74]. As in acne vulgaris, innate AMPs are upregulated in keratinocytes and sebocytes, which may exacerbate the inflammatory response, and alternative treatments resorting to AMPs have become excellent candidates to overcome the limitations faced by antibiotic-based therapies. AMPs can inhibit the growth of *C. acnes* while increasing anti-inflammatory cytokine secretion. The ability of peptides derived from granulysin to decrease the presence of acne in the body, by displaying bactericidal and anti-inflammatory effects against *C. acnes*, has proven just that [75]. Further, the cecropin A-magainin 2 hybrid analog P5 AMP can bind to lipoteichoic acid, lowering *C. acnes*-triggered TLR2 upregulation in keratinocytes and inhibiting microbial growth [76]. Analogous findings were achieved with melittin peptides [77]. Designed peptides which are chemically synthesized are another option to modulate immune responses and impart antibiotic properties against pathogens. Woodburn et al. uncovered five new AMP sequences with bactericidal effectiveness against *C. acnes* commercial strains and clinical isolates. Their specificity for eliminating multidrug-resistant *C. acnes* over mammalian cells was also evidenced. Moreover, the topical administration of the most successful designed AMP was seen to eliminate infection [78].

Oxidative stress is another biproduct of the sebum produced from damaged follicular walls of sebaceous glands in acne. This is initiated by reactive oxygen species (ROS) responsible for the skin irritation associated with acne infection. As most synthetic antioxidant compounds tend to aggravate this situation, acting on the skin microbiota, plant-derived molecules endowed with antimicrobial features have become an important alternative. Vora et al. reported the potentiality of methanolic extracts of *Rosmarinus officinalis*, *Acacia nilotica*, and *Azadirachta indica* to fight infections caused by *C. acnes* while highlighting their antioxidant mechanisms [79]. Resveratrol, extracted from grape seeds, has been incorporated in a carboxymethylcellulose-based gel to serve as a mask with equal purposes [80]. *Rhodomyrtus tomentosa*, from the *Myrtaceae* family, contains various phytochemical compounds (ellagitannins, stilbenes, anthocyanins, flavonols, and phenolic acids) with potent antioxidant, anti-inflammatory, and antimicrobial properties. Recent reports have shown that this natural extract possesses microbiota-regulating agents, which reduce retentional and inflammatory acne lesions while decreasing the abundance of *C. acnes* bacteria [81]. Similar observations were made with TTO and rosemary EOs. Data reported bacteriostatic and bactericidal activity of these two natural compounds against antibiotic-resistant acne clinical isolates [82]. The synergistic effect between EOs has also been examined by Bunrathep et al., who reported an optimal ratio concentration between citronella, lemongrass, and patchouli EOs for an effective antibacterial action against *C. acnes* and *S. epidermidis* [83].

### 4.5. Tinea

Tinea is the name of a group of superficial skin infections caused by fungi, namely, by dermatophytes (filamentous fungi). Depending on the affected region, different designations may be employed: tinea corporis, which affects the trunk, neck, arms, and legs; tinea cruris, which involves the groin region; tinea pedis, commonly found on the feet; tinea faciei, found on the face; tinea capitis, frequently found on the scalp; and tinea manuum, which can be detected in the hands. Regardless of the type, the most common dermatophytes acting to cause infection belong to the genera Trichophyton, Epidermophyton, and Microsporum and have been classified based on their spread mechanism in anthropophilic (human), zoophilic (animal), and geophilic (soil) environments [84,85]. Dermatophytes are capable of digesting keratinized tissues into oligopeptides or amino acids by proteases, serine-subtilisins, and fungolysin. They also reveal potent immunogenic actions by impairing the functions of Th17 cells and reducing secretion of IL-17 and IL-22, thus rendering infections more persistent. In microenvironments of the body characterized by excessive heat, alkaline pH, maceration, or a high relative humidity, proliferation of these pathogens increases significantly [86].

Most treatments for dermatophyte-induced infections resort to topical and oral antifungal preparations. The most employed topical antifungals are based on clotrimazole, ketoconazole, miconazole, naftifine, and terbinafine formulations. Oral therapies are required in more widespread infections in which topical treatments are infective and resort to terbinafine, itraconazole, fluconazole, or griseofulvin courses [85]. Yet, as topical mediations tend to present superior pharmacokinetics, combinations of topical and oral therapies have been highlighted as preferrable in imparting better microbial clearance. Shah et al. proposed the combination of terbinafine, a drug responsible for inhibiting the enzyme squalene epoxidase and, consequently, ergosterol synthesis, with ciclopirox olamine, a topical hydroxypyridone derivative which acts through the chelation of polyvalent metal cations, inhibiting metal-dependent enzymes. Their combination was seen to be very effective against tinea corporis and tinea cruris infections, without conveying detectable side effects to the patients [87].

AMPs are not as common as antifungals in the fight against dermatophyte-derived infections. However, there have already been reports on the effective use of psoriasin, an AMP isolated from psoriatic lesions, against *Trichophyton mentagrophytes*, *Microsporum canis*, and *Epidermophyton floccosum* fungi. This compound is capable of interfering with zinc homeostasis, whose sequestration could result in an important antimicrobial mechanism [88]. Synthetic AMPs have also been uncovered. Lima et al. reported the synthesis of six synthetic AMPs with capability to either be used as models for novel antidermatophytic drugs or as complementary therapies for pre-existing ones. It was seen that the six AMPs were capable of reducing the growth of *T. mentagrophytes* and *Trichophyton rubrum* fungi by up to 95%, by causing severe damage to their hyphal morphology, namely, by disturbing the cell wall, inducing content leakage and consequent cell lysis. Most importantly, these AMPs were far more effective than the conventional antifungals griseofulvin and itraconazole [89]. Alternative strategies by means of plant extracts have also been used in successful topical therapies. Sixty-five single EOs and twenty-one blends have been examined for their mechanisms of action against dermatophytes. From those, cassia, cilantro, cinnamon, thyme, and oregano EOs were found to be the most effective when used individually, while synergistic performances were observed when combining oregano, cilantro, cassia, or cinnamon, and with rosa and cassia formulations [90]. Macrocarpal C, extracted from the leaves of *Eucalyptus globulus Labill*, has shown a significant effect against *T. mentagrophytes* and *T. rubrum* fungi [91]. The crude leaf extract of *Tetradenia riparia* was examined against three clinical isolates of dermatophytes, *T. tonsurans*, *T. mentagrophyte*, and *M. audouinii*, revealing significant antidermatophytic activity [92]. Similar effects were obtained with calyx leaves of *Hibiscussabdariffa L.* when examined against the same pathogens, demonstrating great inhibitory performance against skin isolates due to their alkaloid, tannin, kalekoside, and phenol components [93].

### 4.6. Wound- and Burn-Derived Infections

From a microbial viewpoint, the main purpose of integral, undamaged skin is to avoid the incursion and colonization of underlying tissues. However, the exposure of the subcutaneous layers of the skin following loss of integrity may provide conditions conducive to microorganisms’ growth. In acute wounds, the presence of microbial cells is not significant, with the healing cascade being capable of progressing without constraints. In chronic wounds, however, the presence of polymicrobial and/or resistant infection agents associated with a persistent inflammatory state frequently demands complementary antimicrobial therapies. Often disguised as a comorbidity condition, chronic wounds have been classified as a silent epidemic that affects a large fraction of the world population, with significant economic costs associated [12,17,20]. Similarly, burn wounds represent a susceptible site for opportunistic pathogens’ colonization. Indeed, it is estimated that most registered deaths following burn injuries are correlated with infections rather than osmotic shock or hypovolemia. As microorganisms bind to these wounds, they start penetrating the burn eschar, quickly invading viable subeschar tissue and rendering it infected [94].

Non-healing wounds such as chronic or large-area burns may impair the physiological functions of the skin and spark morbidity or mortality risks. A major advance in treating these infected wounds has been the understanding that the mere presence of microorganisms is less important than their growth rate. Here, *S. aureus* and *Pseudomonas aeruginosa* bacteria have been identified as the most recurrent pathogens. *S. aureus* is typically located in the skin superficial layers, while *P. aeruginosa* is spotted in the innermost regions of the wound bed [95,96]. By controlling infection, antimicrobial agents reduce the tendency for conversion of partial thickness wounds into full thickness wounds. Topical antimicrobial agents based on silver compounds, such as silver nitrate, silver sulfadiazine, mafenide acetate, or nanocrystalline silver, are frequently employed to prevent the spread of bacteria and even fungi (e.g., *C. albicans*) in infected wounds. The silver inhibitory effect results from its powerful interaction with the thiol radicals found in the respiratory enzymes of the microbial cells [97]. Mupirocin, an antibiotic produced from the fermentation process of *Pseudomonas fluorescens*, has also been highlighted as a powerful inhibitory effector against Gram-positive skin flora bacteria, such as coagulase-negative staphylococci and *S. aureus*, including MRSA [98]. Nystatin, an antibiotic produced from *Streptomyces noursei*, is considered a potent antifungal agent, whose action arises from its connection to the cell membrane sterols of fungi, imparting significant effects on wounds colonized by *C. albicans* [99]. Antiseptic agents such as povidone, cadexomer iodine, chlorhexidine, polyhexamethyl biguanide, and honey have also been applied to treat chronic wounds both to reduce inflammation and/or fight infections [17].

The healing significance of natural AMPs in wounds has been extensively analyzed [96]. Yet, in infection-directed therapies, only those AMPs that exhibit reduced cytotoxicity, great stability in microbial-colonized environments, and a broad antimicrobial spectrum of action can be used. The natural-origin peptide LL37 has shown significant effects against *S. aureus* and *S. epidermidis* bacteria, above conventional antibiotics (e.g., vancomycin), while functionalized onto poly(vinyl alcohol)/cellulose acetate dressings [20]. The novispirin G10 peptide revealed a quick action against *P. aeruginosa* on partial thickness burns before being inactivated by proteases [100]. In its turn, PXL150 demonstrated powerful antimicrobial actions by altering the potential of the *S. aureus* membrane and quickly eliminating *P. aeruginosa* bacteria [101]. Dual AMP combinations have also been attempted with pexiganan and nisin peptides. Gomes et al. reported their symbiotic effect against co-cultures of *S. aureus* and *P. aeruginosa* clinical strains and disclosed the need for smaller AMP concentrations, when in blends, to obtain effective outcomes [102].

Natural extracts have been considered, for years, as important players in the treatment of infected wounds [103]. Aloe vera is perhaps one of the most used as it decreases burning, itching, and scarring in many skin infectious diseases. Salicylic acid present in its composition is known to display analgesic and anti-inflammatory effects by hindering prostaglandin production. This effect may be a result of the immunomodulatory assets of the gel polysaccharides present, namely, the acetylated mannans. Aloe vera also displays significant bactericidal and fungicidal effects [104]. Resveratrol, commonly found in grape seeds, and a silent information regulator 1 (SIRT1) agonist, has been known to improve endothelial functions and to possess a strong proangiogenic profile, highly effective against diabetic chronic ulcers [105]. Thymol and tyrosol derived from EOs have been proposed as potential additives to antimicrobial dressings by providing both bactericidal and anti-inflammatory effects [106]. Encapsulated EOs are very common in wound healing [107]. For instance, peppermint EOs loaded into nanostructured lipid carriers have demonstrated important antibacterial effects against a wide range of pathogenic strains, aside from increasing wound contraction, fibroblast penetration, collagen deposition, and re-epithelialization [108]. In another study, cinnamon leaf oil, clove, and cajeput EOs embedded within wet-spun biodegradable microfibers were seen to act more quickly against *S. aureus* than the ampicillin antibiotic [109].

## 5. Conclusions

The response of the skin to microbial pathogen invasion is a highly coordinated phenomena that encompasses all skin layers and, in many cases, the skin microbiota. At a molecular level, the microbiota-produced antimicrobial factors, the host AMPs, and the antimicrobial lipids play a crucial role in fighting pathogenicity. However, the skin, on its own, is not always effective in fighting the penetration of bacteria, fungi, or viruses, and, as such, serious skin infectious may occur. Antimicrobial biomolecules, including antibiotics, AMPs, or plant extracts, may assist in overcoming this limitation. Their propensity to both control infections, eliminating pathogenic microorganisms, and be involved in important anti-inflammatory actions has rendered these biomolecules essential in medicine. Indeed, while inducing infection-solving immunity and regulating innate immunity, these antimicrobial biomolecules dampen and potentially damage pro-inflammatory reactions. Their varied roles and synergisms with innate substrates have been highlighted in the treatment of many skin infectious diseases. This review explored conventional and more recent therapeutic strategies based on active biomolecules to treat some of the most recurrent skin infections. Even though many do not consider skin infections a global problem and do not demand urgent solutions, modern research continues to dedicate its resources to finding more effective strategies that present the smallest impact on human health or our natural reserves.

## Figures and Tables

**Table 1:** Antibiotics most frequently employed to treat psoriasis, their targeted bacteria, mechanisms of action, and effects on the condition (adapted from [58]). (CC BY 4.0 license).

Antibiotic	Bacterial Target	Mechanism	Effect on Psoriasis
**Amoxicillin**	G(+): streptococci, staphylococci, *Enterococcus faecalis*. G(−): *Escherichia coli*, *Helicobacter pylori*, etc.	Release of pro-inflammatory cytokines by macrophages.	Induces generalized pustular psoriasis.
**Vancomycin**	G(+): enterococci, streptococci, *S. aureus* (MRSA), *S. epidermidis*.	Dysregulates gut and skin microbiota; activation of T cells.	Increases the susceptibility of plaque psoriasis.
**Erythromycin**	G(+): streptococci, *Corynebacterium* spp., *S. aureus*, etc. G(−): *Legionella pneumophila*, *Neisseria gonorrhoeae, Chlamydia trachomatis*, etc.	Reduces neutrophil activity; inhibits the action of the cytokines TNFα, IL-6, and IL-8.	Improves plaque psoriasis.
**Azithromycin**	G(+): streptococci, *S. aureus*. G(−): *N. gonorrhoeae*, *Haemophilus* spp., *Moraxella catarrhalis*, etc.	Restores keratinocyte differentiation and decreases hyper-proliferation; reduces cytokines’ action.	Improves plaque psoriasis.
**Tetracyclines**	G(+): streptococci, *S. aureus*, *Listeria monocytogenes*, *Bacillus anthracis*, etc. G(−): *bacilli* spp.	Increases the photosensitivity of the skin; influences the arachidonic acid system.	Exacerbates or induces plaque psoriasis.
**Rifampin**	G(+): *S. aureus* (MRSA), *S. epidermidis*. G(−): *Haemophilus influenza*, *Neisseria meningitidis*.	Supresses T-lymphocytes; inhibits TNFα; increases secretion of IL-6 and IL-10.	Improves plaque psoriasis.
Abbreviations: G(+)—Gram-positive; G(−)—Gram-negative; MRSA—methicillin-resistant *S. aureus.*			

## Data Availability

Not applicable.
